# PI3Kα isoform-dependent activation of RhoA regulates Wnt5a-induced osteosarcoma cell migration

**DOI:** 10.1186/s12935-017-0396-8

**Published:** 2017-02-14

**Authors:** Ailiang Zhang, Ting Yan, Kun Wang, Zhihui Huang, Jinbo Liu

**Affiliations:** 1 Spine Surgery, Third Affiliated Hospital of Soochow University, Changzhou, 213003 Jiangsu China; 2 Safety Assessment and Research Center for Drug, Pesticide and Veterinary Drug of Jiangsu Province, School of Public Health, Nanjing Medical University, Nanjing, 211166 Jiangsu China

**Keywords:** RhoA, Wnt5a, PI3K, Akt, Osteosarcoma, Migration

## Abstract

**Background:**

We have reported that the phosphatidylinositol-3 kinase (PI3K)/Akt signaling pathway mediated Wnt5a-induced osteosarcoma cell migration. However, the signaling pathways regulating Wnt5a/PI3K/Akt-mediated cell migration remains poorly defined in osteosarcoma cells.

**Methods:**

We evaluated the activations of RhoA, Rac1 and Cdc42 in osteosarcoma MG-63 and U2OS cells with small G-protein activation assay. Boyden chamber assays were used to confirm the migration of cells transfected indicated constructs or siRNA specific against RhoA. A panel of inhibitors of PI3K and Akt treated osteosarcoma cells and blocked kinase activity. Western blotting and RhoA activation assay were employed to measure the effect of kinase inhibitors and activations of RhoA and Akt.

**Results:**

We found that Wnt5a had a potent stimulatory effect on RhoA activation, but not on Rac1 and Cdc42 activations. Wnt5a-induced cell migration was largely abolished by siRNA specific against RhoA. DN-RhoA (GFP-RhoA-N19) was also capable of retarding Wnt5a-induced cell migration, but the overexpression of CA-RhoA (GFP-RhoA-V14) was not able to accelerate cell migration. The Wnt5a-induced activation of RhoA was mostly blocked by pretreatment of LY294002 (PI3K inhibitor) and MK-2206 (Akt inhibitor). Furthermore, we found that the Wnt5a-induced activation of RhoA was mostly blocked by pretreatment of HS-173 (PI3Kα inhibitor). Lastly, the phosphorylation of Akt (p-Ser473) was not altered by transfection with siRNA specific against RhoA or DN-RhoA (GFP-RhoA-N19).

**Conclusions:**

Taken together, we demonstrate that RhoA acts as the downstream of PI3K/Akt signaling (specific PI3Kα, Akt1 and Akt2 isoforms) and mediated Wnt5a-induced the migration of osteosarcoma cells.

**Electronic supplementary material:**

The online version of this article (doi:10.1186/s12935-017-0396-8) contains supplementary material, which is available to authorized users.

## Background

The integrity and dynamics of the actin cytoskeleton is a key point for cancer cell migrating into adjacent tissues and leading to the cancer metastasis in the distance [1, 2]. Actin filaments underlie the plasma membrane of mammalian cells, including cancer cells, providing strength and shape to its thin lipid bilayer [3]. Regulation of the dynamic behavior and assembly of the actin filaments allows cancer cells to build an enormous range of structures [4]. There is still largely unknown about the molecular mechanisms underlying the dynamics of the actin cytoskeleton in cancer metastasis, especially for osteosarcoma being characterized by a high malignant and metastatic potential.

In vertebrates, the small GTPase Rho regulates the formation and rearrangement of actin filaments, and have been implicated in the control of cell motility and invasion [5–7]. Rho GTPases are activated and modify the actin cytoskeleton during *Xenopus* embryogenesis and neurite retraction of mouse neuroblastoma cells [8, 9]. Rac1, Cdc42 and RhoA have been shown to play vital roles in growth factor- or cytokine-induced chemotaxis in fibroblasts, macrophages and neutrophils [10–12]. There is also substantial evidence that activation of Rac1, Cdc42 and RhoA is necessary for the metastatic behavior of cancer cells [12, 13]. However, it is still much uncertainty regarding the signaling pathways trigger Rho proteins to regulate metastatic behavior of cancer cells.

The noncanonical Wnt signaling regulates several developmental and oncogenic processes in both insects and vertebrates [14]. Wnt5a has been originally classified into the noncanonical Wnt signaling. The Wnt5a pathways are classified into the following categories for clarity and simplicity: (1) Wnt5a/planar cell polarity signaling; (2) Wnt5a/Ca^2+^ signaling; (3) Wnt5a-RAP1 signaling; (4) Wnt5a/receptor tyrosine kinase-like orphan receptor 2 (ROR2) signaling; (5) Wnt5a/protein kinase A signaling; (6) Wnt5a/GSK3β signaling; (7) Wnt5a/atypical protein kinase C (PKC) signaling; (8) Wnt5a/receptor-like tryosine kinase signaling; and (9) Wnt5a/mammalian target of rapamycin signaling [15]. In the previous study, we found that the PI3K/Akt signaling pathway mediated Wnt5a-induced osteosarcoma cell migration. Here, we demonstrates that RhoA acts as the downstream of PI3K/Akt signaling and mediated Wnt5a-induced the migration of osteosarcoma cells.

## Methods

### Cell culture

Human MG-63 and U2OS osteosarcoma cell lines were purchased from Cells Resource Center of Shanghai Institutes for Biological Sciences, Chinese Academy of Sciences (Shanghai, China). These cells were cultured in Dulbecco-modified Eagle’s medium (DMEM) supplemented with 10% fetal bovine serum (FBS, Hyclone, Logan, UT), at 37 °C in a humidified atmosphere with 5% CO_2_. MG-63 cells were plated onto 6-well cell culture clusters (Costar) and grown to 80% confluence, and then serum-starved for 24 h. These cells were subsequently treated with recombinant sfrp2 or Wnt5a (R&D Systems, Minneapolis, MN) or PI3K/Akt inhibitors (LY294002, HS-173, TGX-221, CZC24832, CAL-101, MK-2206, A-674563, CCT128930) (Selleck, Houston, TX) before small G-protein activation assays and cell migration assays.

### Plasmids and small interfering RNA (siRNA)

The constructs GFP-RhoA-N19, GFP-RhoA-V14 and vectors were kindly provided by Dr. Zhu (Nanjing Medical University, China). RhoA constructs or siRNA duplexes specific for RhoA (Santa Cruz Biotechnology, Santa Cruz, CA) were transiently transfected into MG-63 and U2OS cells by using Lipofectamine 2000 reagent (Invitrogen, Carlsbad, CA) in serum-free OPTI-MEM according to the manufacturer’s instructions. The cells were switched to fresh medium containing 10% FBS 6 h after the transfection and cultured for 48 h. The cells transfected with RhoA constructs or siRNA were used for analyzing the expression of these proteins and cell migration.

### Small G-protein activation assay

For RhoA, Cdc42 and Rac1 activation assays, MG-63 and U2OS cells were seeded into 6-well plates and treated with Wnt5a (100 or 200 ng/mL) or indicated PI3K/Akt inhibitors [16]. The experiments were then performed according to the manufacturer’s protocol (Cytoskeleton Inc., Denver, CO, USA). The activation of RhoA, Cdc42 or Rac1 was normalized to the control group (cells treated with vehicle). RhoA, Cdc42 and Rac1 activation assays were performed in triplicate.

### Cell migration assays

Cell migration was assessed in a modified Boyden chamber (Costar), in which two chambers were separated by a polycarbonate membrane (pore diameter, 8.0 μm). MG-63 and U2OS cells transfected with RhoA constructs or siRNA were grown to subconfluence in 6-well plates and then detached. Thereafter, they were centrifuged and rendered into single cell suspensions in serum-free culture medium supplemented with 5 μg/mL BSA. The suspensions containing 5000 cells were added to wells with a membrane placed in the bottom. Medium containing Wnt5a or vehicle was added to the upper and lower compartment of the Boyden chamber. The cells were allowed to migrate for 4 h at 37 °C in this assay. Thereafter, the medium was discarded, stationary cells were removed with a cotton-tipped applicator and the membranes were cut out of the chamber and stained with 0.5% crystal violet. The response was evaluated in a light microscope (Nikon, Tokyo, Japan) by counting the number of cells that had migrated into the membrane.

### Western blotting

Subconfluent cells were washed twice with PBS, and then lysed with ice-cold RIPA lysis buffer (50 mmol/L Tris, 150 mmol/L NaCl, 1% Triton X-100, 1% sodium deoxycholate, 0.1% SDS, 1 mmol/L sodium orthovanadate, 1 mmol/L sodium fluoride, 1 mmol/L EDTA, 1 mmol/L PMSF, and 1% cocktail of protease inhibitors) (pH7.4). The lysates were then clarified by centrifugating at 12,000*g* for 20 min at 4 °C. The protein extracts were separated by SDS-PAGE. The immunoblotting procedure was performed as described [17] and the following antibodies were used: rabbit anti-Akt antibody, rabbit anti-phospho-Akt (p-Ser473) antibody (Cell Signaling Technology, Danvers, MA). Protein bands were detected by incubating with horseradish peroxidase-conjugated antibodies (Santa Cruz Biotechnology, Santa Cruz, CA) and visualized with ECL reagent (Thermo Scientific, Rockford, IL). The gray values were taken by Tanon imaging analysis system (Tanon, Shanghai, China).

### Statistical analysis

All experiments here were repeated at least three times, with independent treatments, each of which showed essentially the same results. The data were analyzed using Student’s *t* test by SPSS statistical software package. All the results were expressed as mean ± SD. For all analyses a two-sided *p* < 0.05 was deemed statistically significant.

## Results

### RhoA activation was stimulated by Wnt5a treatment in osteosarcoma cells

**Fig. 1:**
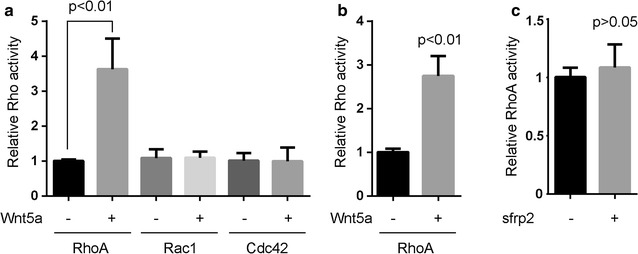
Wnt5a triggers RhoA activation of osteosarcoma cells. (**a** and **b**) Human osteosarcoma cells MG-63 (**a**) and U20S (**b**), serum-deprived for 24 h, were untreated or treated with 100 ng/mL of Wnt5a and harvested at 30 min after the start of treatment for small G-protein activation assays. Data were presented as mean ± SD of 3 determinations. The relative Rho activity was normalized to the average value of Wnt5a-untreated group. **c** Serum-deprived MG-63 cells were pre-treated with 1 µg/mL sfrp2 (an antagonist that directly binds to Wnt5a) for 1 h, then were incubated with 100 ng/mL Wnt5a and harvested at 30 min after the start of Wnt5a treatment. Data were presented as mean ± SD of 3 determinations. The relative RhoA activity was normalized to the average value of sfrp2-untreated group

To assess the effect of Wnt5a on Rho activation in human osteosarcoma cells, we treated MG-63 and U2OS cells with 100 or 200 ng/mL Wnt5a, and measured the Rho activation by small G-protein activation assay. We found that Wnt5a had a potent stimulatory effect on RhoA activation, but not on Rac1 and Cdc42 activations (Fig. 1a, b; Additional file 1: Figure S1). Sfrp2, an antagonist directly binding to Wnt5a, was significantly blocked the stimulatory effect of Wnt5a on RhoA activation (Fig. 1c). Thus, RhoA activation is regulated by Wnt5a signaling in MG-63 osteosarcoma cells.

### RhoA mediated the Wnt5a-induced cell migration

**Fig. 2:**
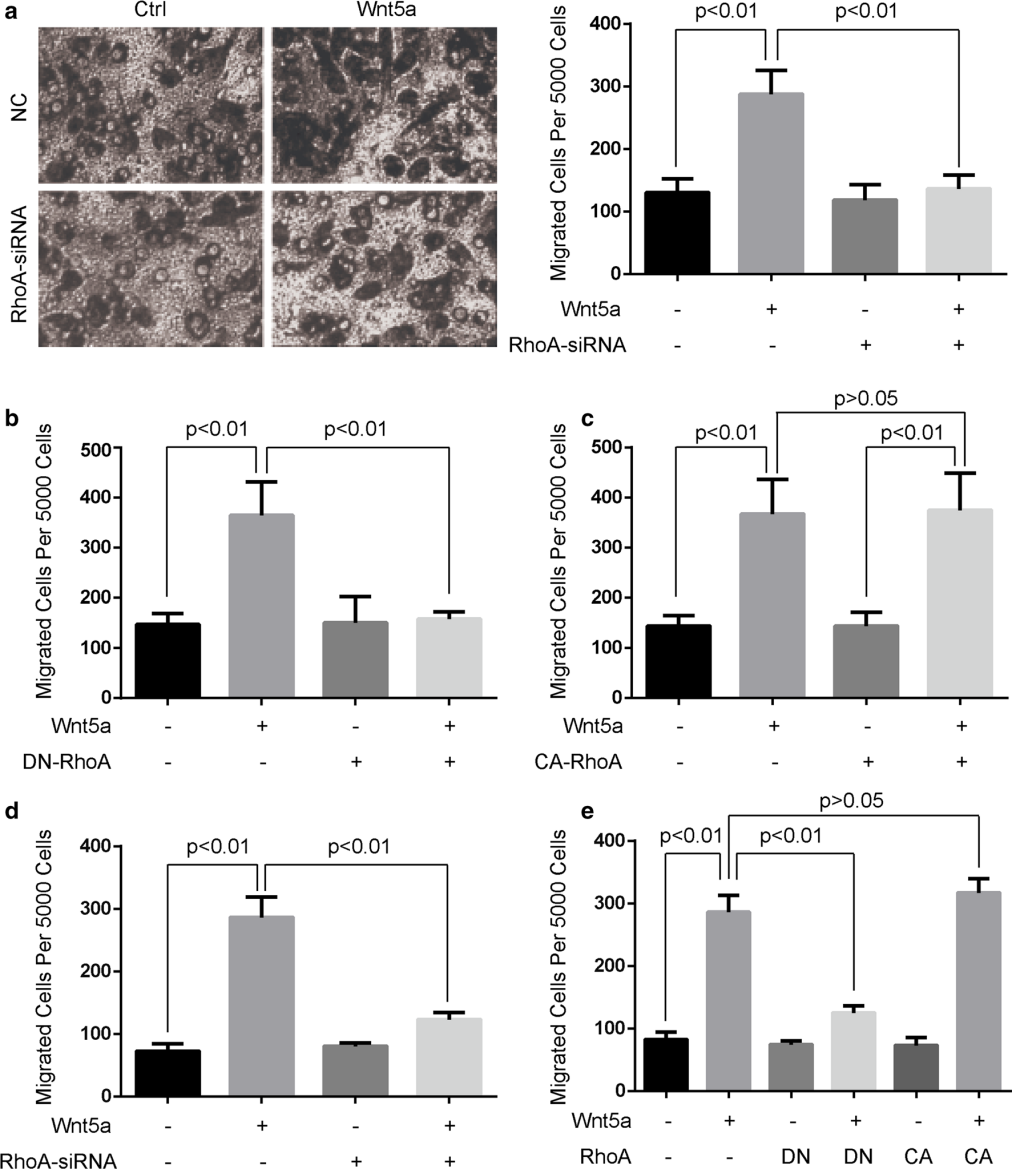
RhoA activation is required for Wnt5a-induced cell migration. Cell migration rate was determined by using Boyden chamber assays in MG-63 and U20S cells incubated in the absence (0 ng/mL) or presence of 100 ng/mL Wnt5a for 4 h. **a**, **d** MG-63 (**a**) and U20S cells (**d**) were transfected with RhoA-siRNA or scrambled siRNA (NC), then subjected to Boyden chamber assays. **b**, **e** MG-63 (**b**) and U20S cells (**e**) were transfected with GFP-RhoA-N19 (DN-RhoA) or vector, then subjected to Boyden chamber assays. **c**, **e** MG-63 (**c**) and U20S cells (**e**) were transfected with GFP-RhoA-V14 (CA-RhoA) or vector, then subjected to Boyden chamber assays. All data were presented as mean ± SD of 3 determinations

The finding that Wnt5a could induce RhoA activation in MG-63 cells prompted us to determine whether RhoA activation was required for Wnt5a-mediated cell migration. Wnt5a-induced cell migration was largely abolished by siRNA specific against RhoA (Fig. 2a, d), suggesting that RhoA activation is required for Wnt5a-induced migration of MG-63 and U2OS cells. We also used dominant-negative constructs (DN-RhoA) to knock down RhoA expression in osteosarcoma cells and checked whether Wnt5a-induced cell migration could be inhibited. DN-RhoA (GFP-RhoA-N19) was capable of retarding Wnt5a-induced cell migration (Fig. 2b, e), but the overexpression of CA-RhoA (GFP-RhoA-V14) was not able to accelerate cell migration (Fig. 2c, e). These findings suggest that RhoA mediates the Wnt5a-induced cell migration of osteosarcoma cells.

### PI3Kα was required for Wnt5a-induced RhoA activity in osteosarcoma cells

**Fig. 3:**
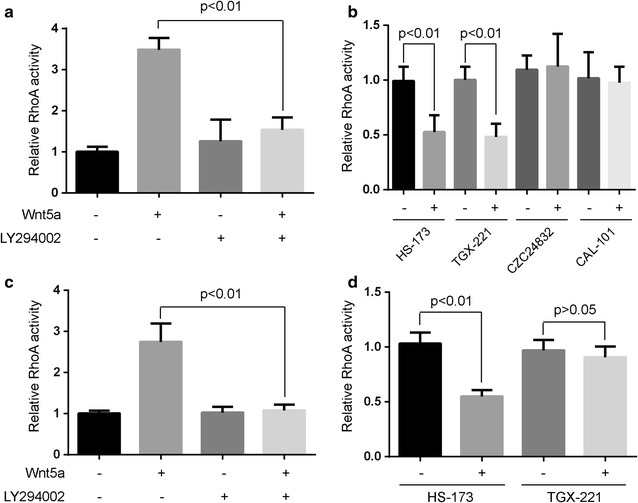
PI3 Kα isoform is required for Wnt5a-induced RhoA activity. **a**, **c** Human osteosarcoma cells MG-63 (**a**) and U20S (**c**), serum-deprived for 24 h, were pre-treated with 10 µmol/L LY294002 (PI3K inhibitor) for 1 h, were incubated with 100 ng/mL Wnt5a and harvested at 30 min after the start of Wnt5a treatment. The relative RhoA activity was normalized to the average value of LY294002/Wnt5a-untreated group. **b**, **d** Serum-deprived MG-63 (**b**) and U20S cells (**d**) were pre-treated with 1 nmol/L HS-173 (PI3Kα inhibitor), 10 nmol/L TGX-221 (PI3Kβ inhibitor), 10 nmol/L CZC24832 (PI3Kγ inhibitor) and 10 nmol/L CAL-101 (PI3Kδ inhibitor) for 1 h, then were incubated with 100 ng/mL Wnt5a and harvested at 30 min after the start of Wnt5a treatment. Data were presented as mean ± SD of 3 determinations. The relative RhoA activity was normalized to the average value of each inhibitor-untreated group

To state the involvement of PI3K in Wnt5a-induced activation of RhoA, we tested the effect of PI3K inhibitors for RhoA activation. Human osteosarcoma cells, pretreated with 10 μmol/L LY294002 (PI3K inhibitor) for 1 h, were incubated with 100 ng/mL of Wnt5a. The cells were harvested 30 min after the start of Wnt5a treatment and the cell lysates were subjected to small G-protein activation assay. The Wnt5a-induced activation of RhoA was mostly blocked by pretreatment of LY294002 (Fig. 3a, c).

Moreover, we want to know which isoforms of PI3K regulate the Wnt5a-induced activation of RhoA. MG-63 and U2OS cells were pretreated with 1 nmol/L HS-173 (PI3Kα inhibitor), 10 nmol/L TGX-221 (PI3Kβ inhibitor), 10 nmol/L CZC24832 (PI3Kγ inhibitor) and 10 nmol/L CAL-101 (PI3K**δ** inhibitor), respectively, then were incubated with 100 ng/mL Wnt5a. The Wnt5a-induced activation of RhoA was mostly blocked by pretreatment of HS-173 and TGX-221 in MG-63 cells, respectively (Fig. 3b). However, Wnt5a-induced activation of RhoA was only blocked by pretreatment of HS-173 in U2OS cells (Fig. 3d). These data indicate that PI3Kα mediates Wnt5a-induced activation of RhoA in osteosarcoma cells.

### Akt1 and Akt2 were required for Wnt5a-induced RhoA activity in osteosarcoma cells

**Fig. 4:**
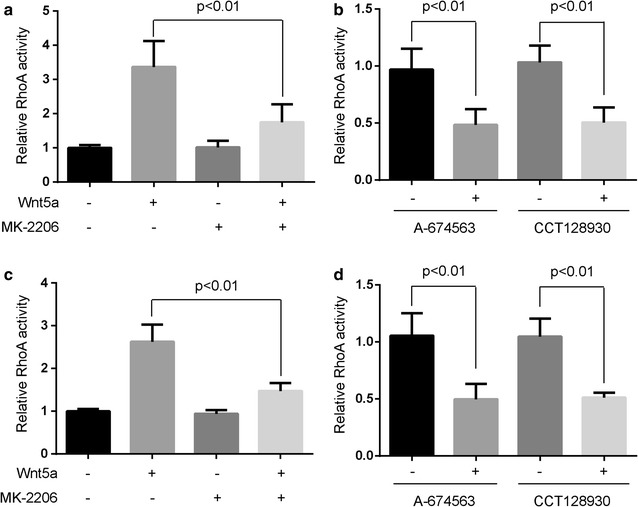
Akt1 and Akt2 isoforms are required for Wnt5a-induced RhoA activity. **a**, **c** Human osteosarcoma cells MG-63 (**a**) and U20S (**c**), serum-deprived for 24 h, were pre-treated with 10 nmol/L MK-2206 (Akt inhibitor) for 1 h, were incubated with 100 ng/mL Wnt5a and harvested at 30 min after the start of Wnt5a treatment. The relative RhoA activity was normalized to the average value of LY294002/Wnt5a-untreated group. **b**, **d** Serum-deprived MG-63 (**b**) and U20S cells (**d**) were pre-treated with 10 nmol/L A-674563 (Akt1 inhibitor) and 10 nmol/L CCT128930 (Akt2 inhibitor) for 1 h, then were incubated with 100 ng/mL Wnt5a and harvested at 30 min after the start of Wnt5a treatment. Data were presented as mean ± SD of 3 determinations. The relative RhoA activity was normalized to the average value of each inhibitor-untreated group

To demonstrate the involvement of Akt in Wnt5a-induced activation of RhoA, we tested the effect of Akt inhibitors for RhoA activation. Osteosarcoma cells, pretreated with 10 nmol/L MK-2206 (Akt inhibitor) for 1 h, were incubated with 100 ng/mL of Wnt5a. The cells were harvested 30 min after the start of Wnt5a treatment and the cell lysates were subjected to small G-protein activation assay. The Wnt5a-induced activation of RhoA was mostly blocked by pretreatment of MK-2206 (Fig. 4a, c).

Moreover, we want to know which isoforms of Akt regulate the Wnt5a-induced activation of RhoA. MG-63 and U2OS cells were pretreated with 10 nmol/L A-674563 (Akt1 inhibitor) and 10 nmol/L CCT128930 (Akt2 inhibitor), respectively, then were incubated with 100 ng/mL Wnt5a. The Wnt5a-induced activation of RhoA was mostly blocked by pretreatment of A-674563 and CCT128930, respectively (Fig. 4b, d). These data indicate that Akt1 and Akt2 mediate Wnt5a-induced activation of RhoA in osteosarcoma cells.

### RhoA acted as the downstream of Wnt5a/PI3K/Akt signaling

**Fig. 5:**
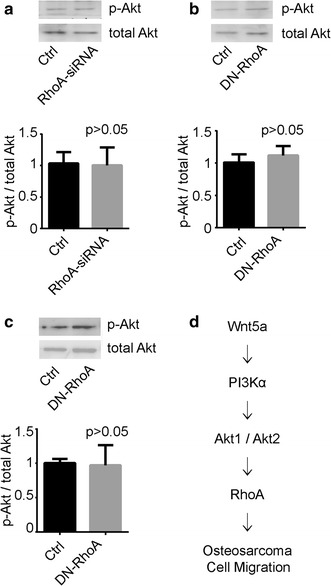
RhoA acts as the downstream of Wnt5a/PI3K/Akt signaling. **a** Human osteosarcoma cells MG-63 were transfected with RhoA-siRNA or scrambled siRNA (Ctrl) for 48 h, then were treated with 100 ng/mL of Wnt5a and harvested at 30 min after the start of Wnt5a treatment for SDS-PAGE and immunoblot analysis with antibodies to p-Akt and total Akt. p-Akt/total Akt ratios were normalized to the average value of Ctrl group. Data were presented as mean ± SD of 3 determinations. **b**, **c** MG-63 (**b**) and U20S cells (**c**) were transfected with DN-RhoA (GFP-RhoA-N19) or vector (Ctrl) for 48 h, then were treated with 100 ng/mL of Wnt5a and harvested at 30 min after the start of Wnt5a treatment for SDS-PAGE and immunoblot analysis. p-Akt/total Akt ratios were normalized to the average value of Ctrl group. Data were presented as mean ± SD of 3 determinations. **d** A schematic illustration of the regulation of RhoA in Wnt5a/PI3K/Akt signaling (See text for detail)

We next examined whether RhoA was downstream of PI3K/Akt in human osteosarcoma cells. MG-63 cells, transfected with RhoA-siRNA, were treated with 100 ng/mL of Wnt5a. The cells were harvested at 30 min after the start of Wnt5a treatment, followed by SDS-PAGE and Western blotting analyses. Akt showed unchanged signs of phosphorylation at Ser473, which represents the Akt activation state, after Wnt5a treatment (Fig. 5a). The same assays were performed to detect the phosphorylated of Akt after DN-RhoA (GFP-RhoA-N19) transfection, the phosphorylation of Akt was also not altered after stimulation with Wnt5a (Fig. 5b, c).

In conclusion, RhoA acts as the downstream of PI3K/Akt signaling (specific PI3KαAkt1 and Akt2 isoforms) and mediated Wnt5a-induced the migration of osteosarcoma cells (Fig. 5d).

## Discussion

Wnt5a, a prototypic ligand that activates a β-catenin independent pathway in Wnt signaling, has been demonstrated to exert differential effects on cancer development [18, 19]. Wnt5a promotes breast cancer cell migration via Dishevelled 2 (Dvl2)/Dishevelled-associated activator of morphogenesis 1 (Daam1)/RhoA signaling pathway [20]. Wnt5a-Ror2 signaling enhances expression of CXCL16 in mesenchymal stem cells (MSCs) and enhanced secretion of CXCL16 from MSCs, leading to the promotion of its proliferation [21]. In the previous study, we have found that Wnt5a induces the migration of MG-63 osteosarcoma cells by triggering PI3K/Akt signaling, suggesting that Wnt5a acts as a migratory stimulator in osteosarcoma cells [16]. Thus, 100 ng/mL Wnt5a was used in study to identify the mechanism whereby changes in the migration of osteosarcoma cells were induced.

PI3Ks are a family of enzymes involved in cellular functions such as cell growth, proliferation, differentiation, motility, survival and intracellular trafficking, at least including four isoforms (PI3Kα, PI3Kβ, PI3Kγ and PI3Kδ) [22]. Akt, also known as protein kinase B (PKB), is the direct target of PI3K, including three isoforms (Akt1, Akt2 and Akt3) [23]. It is still largely unknown which isoforms of PI3Ks and Akts regulate the Wnt5a-induced migration of osteosarcoma cells. Because Akt3 appears to be predominantly expressed in the brain [24], we used the specific inhibitors to block PI3Kα, PI3Kβ, PI3Kγ, PI3Kδ, Akt1 and Akt2 activity and studies the effects of each isoform of PI3Ks and Akts in osteosarcoma cell migration in this study. Here, PI3Kα, PI3Kβ, Akt1 and Akt2 isoforms regulate Wnt5a-induced osteosarcoma cell migration.

RhoA promotes focal adhesion and regulates cell contractility, leading to the cell migration [25]. Wnt3a triggers RhoA activity to regulate neurite retraction of mouse neuroblastoma cells [8]. RhoA is involved in Wnt5a signaling and promotes the migration of MDA-MB-231 breast cancer cells [20]. In gastric cancer cells, down-regulation of PI3K/Akt/GSK3β signaling in SGC-7901 cells suppressed Wnt5a-induced activation of RhoA [17]. In this study, we demonstrated that RhoA activation acts as the downstream of Wnt5a/PI3K/Akt signaling, and is specifically regulated by PI3Kα, Akt1 and Akt2 isoforms in osteosarcoma cells.

## Conclusions

We present the evidence here that RhoA mediates Wnt5a-induced osteosarcoma cell migration via PI3Kα, Akt1 and Akt2 isoforms. These findings elucidate a molecular pathway linking RhoA signaling to Wnt5a/PI3K/Akt in cell motility. This result will contribute to further understanding of biological roles of Wnt5a/PI3K/Akt/RhoA in cell migration of osteosarcoma and other cancers.

## Additional file

**Additional file 1: Figure S1.** Wnt5a does not elevate the activation of Rac1 and Cdc42 in osteosarcoma cells. Human osteosarcoma cells MG-63 and U20S, serum-deprived for 24 h, were untreated or treated with 200 ng/mL of Wnt5a and harvested at 30 min after the start of treatment for small G-protein activation assays. Data were presented as mean ± SD of 3 determinations. The relative Rho activity was normalized to the average value of Wnt5a-untreated group.

## Abbreviations

PI3K: phosphatidylinositol-3 kinase
ROR2: receptor tyrosine kinase-like orphan receptor 2
PKC: atypical protein kinase C
DMEM: Dulbecco-modified Eagle’s medium
FBS: fetal bovine serum
Dvl2: Dishevelled 2
Daam1: Dishevelled-associated activator of morphogenesis 1
MSC: mesenchymal stem cell

## References

[1] Ren L (2009). The actin-cytoskeleton linker protein ezrin is regulated during osteosarcoma metastasis by PKC. Oncogene.

[2] Khanna C (2004). The membrane-cytoskeleton linker ezrin is necessary for osteosarcoma metastasis. Nat Med.

[3] Henty-Ridilla JL (2016). Accelerated actin filament polymerization from microtubule plus ends. Science.

[4] Lehtimaki J, Hakala M, Lappalainen P (2016). Actin filament structures in migrating cells. Handb Exp Pharmacol..

[5] Littlefield R, Almenar-Queralt A, Fowler VM (2001). Actin dynamics at pointed ends regulates thin filament length in striated muscle. Nat Cell Biol.

[6] Genova JL (2000). Functional analysis of Cdc42 in actin filament assembly, epithelial morphogenesis, and cell signaling during Drosophila development. Dev Biol.

[7] Chang E (2011). MK2 SUMOylation regulates actin filament remodeling and subsequent migration in endothelial cells by inhibiting MK2 kinase and HSP27 phosphorylation. Blood.

[8] Tsuji T (2010). Involvement of p114-RhoGEF and Lfc in Wnt-3a- and dishevelled-induced RhoA activation and neurite retraction in N1E − 115 mouse neuroblastoma cells. Mol Biol Cell.

[9] Kim GH, Han JK (2005). JNK and ROKalpha function in the noncanonical Wnt/RhoA signaling pathway to regulate Xenopus convergent extension movements. Dev Dyn.

[10] Chen WD (2013). RhoA-Rho kinase signaling pathway mediates adventitial fibroblasts differentiation to myofibroblasts induced by TGF-beta1. Sheng Li Xue Bao.

[11] Allen WE (1998). A role for Cdc42 in macrophage chemotaxis. J Cell Biol.

[12] Banyard J (2000). Motility and invasion are differentially modulated by Rho family GTPases. Oncogene.

[13] Keely PJ (1997). Cdc42 and Rac1 induce integrin-mediated cell motility and invasiveness through PI(3)K. Nature.

[14] Logan CY, Nusse R (2004). The Wnt signaling pathway in development and disease. Annu Rev Cell Dev Biol.

[15] Zhu N (2014). Challenging role of Wnt5a and its signaling pathway in cancer metastasis (Review). Exp Ther Med.

[16] Zhang A (2014). Wnt5a promotes migration of human osteosarcoma cells by triggering a phosphatidylinositol-3 kinase/Akt signals. Cancer Cell Int.

[17] Liu J (2013). PI3 K/Akt-dependent phosphorylation of GSK3beta and activation of RhoA regulate Wnt5a-induced gastric cancer cell migration. Cell Signal.

[18] Asem, M.S., et al., Wnt5a Signaling in Cancer. Cancers (Basel), 2016. **8**(9). 10.3390/cancers8090079 PMC5040981 27571105

[19] Leris AC (2005). WNT5A expression in human breast cancer. Anticancer Res.

[20] Zhu Y (2012). Dvl2-dependent activation of Daam1 and RhoA regulates Wnt5a-induced breast cancer cell migration. PLoS ONE.

[21] Takiguchi G (2016). Wnt5a-Ror2 signaling in mesenchymal stem cells promotes proliferation of gastric cancer cells by activating CXCL16-CXCR6 axis. Cancer Sci.

[22] Fritsch R, Downward J (2013). SnapShot: class I PI3 K isoform signaling. Cell.

[23] Linnerth-Petrik NM (2016). Akt isoform specific effects in ovarian cancer progression. Oncotarget..

[24] Yang ZZ (2004). Physiological functions of protein kinase B/Akt. Biochem Soc Trans.

[25] Aifuwa I (2015). Senescent stromal cells induce cancer cell migration via inhibition of RhoA/ROCK/myosin-based cell contractility. Oncotarget.

