# Supplementary figures and images for: PI3Kα isoform-dependent activation of RhoA regulates Wnt5a-induced osteosarcoma cell migration
Source: Cancer Cell Int. 2017 Feb 14;17:27. doi: 10.1186/s12935-017-0396-8 (PMC5310072; doi:10.1186/s12935-017-0396-8)

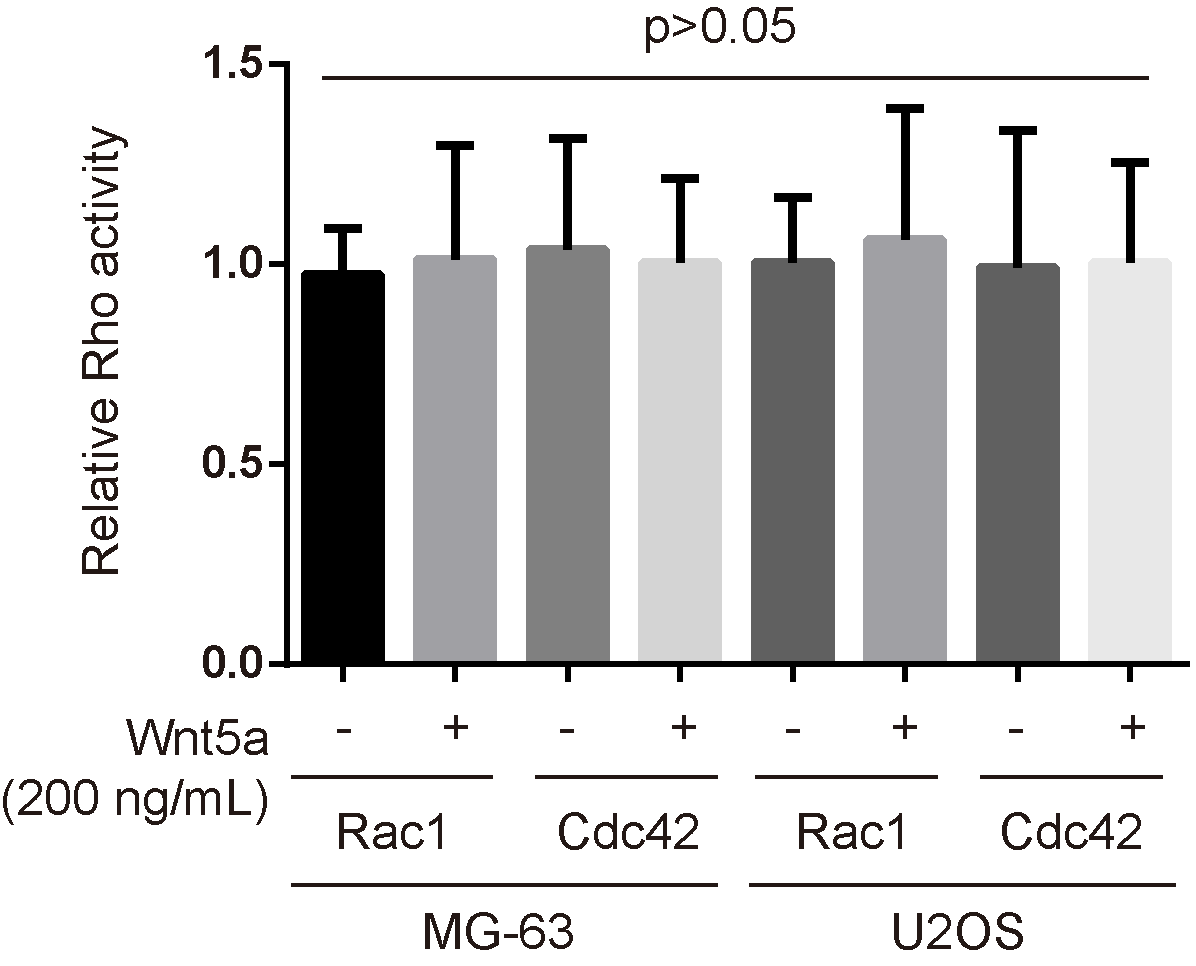

Supplement: Supplementary file 1 — Additional file 1: Figure S1. Wnt5a does not elevate the activation of Rac1 and Cdc42 in osteosarcoma cells. Human osteosarcoma cells MG-63 and U20S, serum-deprived for 24 h, were untreated or treated with 200 ng/mL of Wnt5a and harvested at 30 min after the start of treatment for small G-protein activation assays. Data were presented as mean ± SD of 3 determinations. The relative Rho activity was normalized to the average value of Wnt5a-untreated group. [file 12935_2017_396_MOESM1_ESM.tif]
